# Huntington's Disease with Parkinson-Like Symptoms and Abnormal [ ^123^ I] Ioflupane SPECT-CT (DaTs): A Case Report and Literature Review

**DOI:** 10.1055/s-0045-1812051

**Published:** 2025-10-04

**Authors:** Sarah Amro, Chamani Punchihewa, Edina Bravo, Sabina Dizdarevic

**Affiliations:** 1 Department of Nuclear Medicine, Royal Sussex County Hospital, Brighton, United Kingdom; 2 Department of Nuclear Medicine, University Hospitals Sussex NHS Foundation Trust, Brighton, United Kingdom; 3 Department of Neurology, University Hospitals Sussex NHS Foundation Trust, Brighton, United Kingdom; 4 Department of Nuclear Medicine, Brighton and Sussex Medical School, Brighton, United Kingdom

**Keywords:** Huntington's disease, DaTSCAN, dopamine transporter imaging, neurodegeneration, [ ^123^ I] Ioflupane

## Abstract

Huntington's disease (HD) is an autosomal dominant neurodegenerative disorder caused by an unstable expansion of CAG trinucleotide repeats that lead to progressive degeneration of postsynaptic striatal medium-spiny GABAergic neurons. We report a case of abnormal [ ^123^ I] Ioflupane single-photon emission computed tomography/computed tomography (DaTSCAN) and a subsequent genetic test confirming HD. A 68-year-old man presented with progressive memory difficulties, auditory hallucinations, nightmares, suicidal thoughts, low mood, and involuntary limb movements. DaTSCAN showed borderline reduced putaminal binding ratios bilaterally, more prominent on the left side, and a low putamen-to-caudate ratio suggesting degenerative parkinsonism. Genetic testing revealed one pathogenic expansion (40/17 CAG repeats), confirming HD. Postsynaptic dopaminergic involvement has previously been documented in postmortem studies. However, an emerging in vivo research also suggests presynaptic dopaminergic involvement. While genetic testing remains the gold standard for diagnosis of HD, DaTSCAN may play a role in assessing dopamine transporter activity and tracking the progression of neurodegeneration in HD.

## Introduction

Huntington's disease (HD) is an autosomal dominant disorder caused by an expansion of CAG repeats in the HD gene, leading to a mutant protein accumulation and subsequent degeneration mainly of the postsynaptic striatal medium-spiny GABAergic neurons. It's characterized by a gradual onset of involuntary movements, cognitive difficulties, and behavioral or emotional disturbances. 1 The average age of onset is typically between 30 and 50 years, but cases have been reported from as young as 2 to as old as 85 years. 2 It is diagnosed primarily through genetic testing, which confirms the presence of an expanded CAG repeat in the *HTT* gene. This test is definitive and can identify the disease even before symptoms appear. 1

While genetic testing remains the definitive method for diagnosing HD, nuclear imaging scans can offer valuable insights into the functional and molecular changes in HD. A Dopamine Transporter scan (DaT) is a nuclear imaging scan using single-photon emission computed tomography (SPECT) to visualize dopamine transporter levels in the brain, particularly in the striatum. The tracer used is [ ^123^ I] Ioflupane (radiolabeled cocaine analogue) which binds specifically to dopamine transporters in presynaptic neurons. It is primarily indicated for evaluation of patients with suspected parkinsonian syndromes and for differentiation of presynaptic parkinsonian syndromes from parkinsonism without presynaptic dopaminergic loss. 3

Here, we present a case of a patient with reduced presynaptic dopamine uptake on [ ^123^ I] Ioflupane SPECT-CT (DaTSCAN), with subsequent genetic testing confirming HD.

## Case Report

A 68-year-old right-handed male patient with a past medical history of hypothyroidism and hypertension presented with increased anxiety, depression, auditory hallucinations, nightmares, and suicidal thoughts. He reported that his symptoms began 2 years ago, and he had no prior psychiatric history. His social history was notable for alcohol dependence, abstaining since 1989 and no documented family history for neurodegenerative diseases.

The patient was hospitalized and started on antipsychotics (olanzapine, quetiapine). Over time, his condition progressed, and he began experiencing involuntary movements (jerky limb movements, poor coordination, unsteadiness, multiple falls, orofacial dyskinesia, and foot drop). Antipsychotics were discontinued as they exacerbated these involuntary movements. On neurological examination, findings were largely unremarkable, aside from unsteadiness on the heel-to-toe test, past-pointing in the left arm, and mild dysdiadochokinesia.

A brain magnetic resonance imaging (MRI) showed cerebral and cerebellar hemispheric atrophy. A subsequent DaTSCAN was conducted. The patient was pretreated with a thyroid blocking agent approximately an hour before the intravenous administration of 185 MBq of [ ^123^ I] Ioflupane. Three hours after the administration, SPECT-CT imaging was performed using a dual-head SPECT gamma camera model Symbia Intevo (SIEMENS Healthineers) with integrated CT (16 slices/rotation). Striatal binding ratios were calculated using DaTQuant software by GE Healthcare, to semiquantify striatum to background (occipital region) ratios of SPECT counts within the predefined volume of interests.

**Fig. 1:**
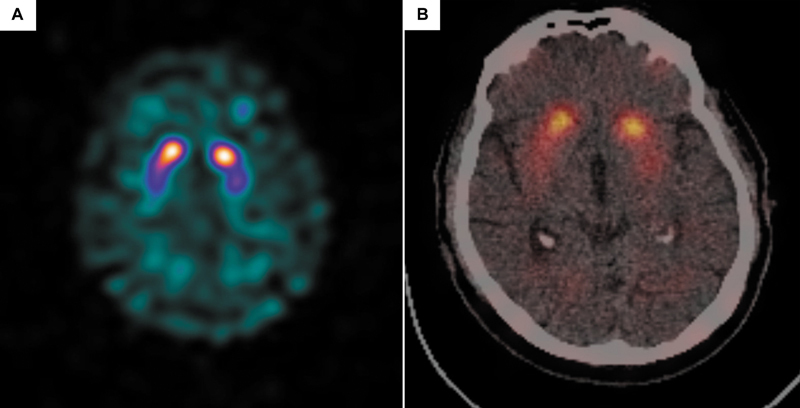
( **A** , **B** ) DaTSCAN SPECT/CT axial images showing slightly reduced uptake in bilateral putamina, left slightly greater than the right. Uptake in the caudate heads seems normal. SPECT/CT, single-photon emission computed tomography–computed tomography.

**Fig. 2:**
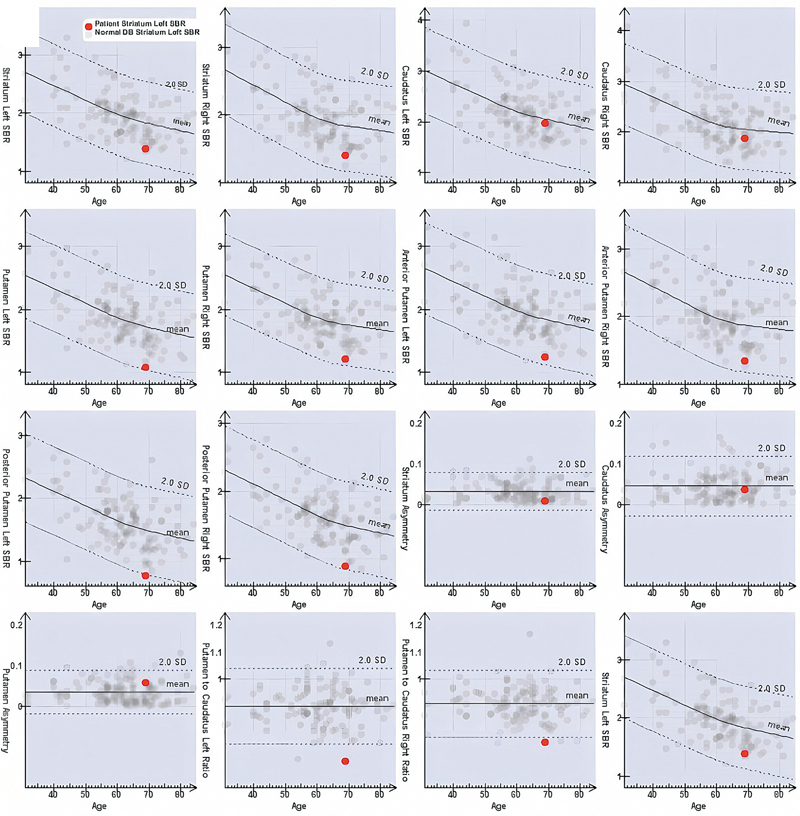
Semiquantitative analysis showing reduced putaminal binding ratios bilaterally (more prominent on the left) with Putamen/Caudate ratio below 2 standard deviation.

DaTSCAN revealed borderline reduced putaminal binding ratios bilaterally (more prominent on the left) and left putamen-to-caudate asymmetry ( Figs. 1A, B ; Fig. 2 ). These findings were suggestive of a dopamine transporter deficit at the presynaptic level, so degenerative parkinsonism (Idiopathic Parkinson’s disease/Parkinsonian syndromes [IPD/PS]) was suspected.

Due to persistent nightmares and involuntary movements, Huntington's disease (HD) was strongly considered. Genetic testing confirmed the diagnosis, revealing one pathogenic allele with 40 (± 1) CAG repeats and one nonpathogenic allele with 17 CAG repeats.

## Discussion

This case presents several diagnostic challenges, including atypical DaTSCAN findings, a lack of documented family history, and the impact of antipsychotic therapy on symptomatology. Postsynaptic dopaminergic involvement has been described in several postmortem and imaging studies. 4 5 6 There is also evolving in vivo imaging evidence of presynaptic dopaminergic involvement. 4 From imaging perspective, several reported cases have shown that HD may present with reduced presynaptic dopamine uptake on DaT imaging ( Table 1 ). 6 7 8 9 10 11 12

**Table 1:** Previous studies reporting abnormal DAT imaging in Huntington's disease

Study ID	Study design	Number of patients	Tracer	CAG repeats	Main findings on DAT imaging	Conclusion
Gamez-2010 7	Observational study	12 HD	[ ^123^ I]-FP-CIT	45.2 (3.7) *mean/SD	8 abnormal visual analysis 4 abnormal semiquantitative analysis	Reduced striatal uptake observed in HD patients suggests pre-synaptic dopaminergic involvement
Hwang-2013 6	Observational study	4 HD 7 controls	[ ^99^ ᵐTc] TRODAT-1 [ ^123^ I] IBZM	17/18 17/49 17/48 17/46	1 Reduced DAT binding 3 Reduced striatal D2 receptor binding	The postsynaptic nigrostriatal pathway is involved, while the presynaptic part is generally unaffected but may be in advanced cases
Kiferle-2013 8	Observational study	12 HD 12 Controls	[ ^123^ I]-FP-CIT	37–47	Striatal, caudate, and putaminal uptake was significantly lower in HD compared with controls	Nigrostriatal degeneration may occur in symptomatic HD but doesn't play a central role in in the pathogenesis of cognitive and motor features
Gamez-2014 9	Observational study	4 HD	[ ^123^ I]-FP-CIT	Not stated	Progressive uptake reduction in the caudate and putamen average annual decline of 5.8% in the caudate and 9.6% in the putamen over a 2-y follow-up	[ ^123^ I]-FP-CIT/SPECT is useful for investigating presynaptic dopaminergic degeneration in HD and may serve as a biomarker for disease progression
Larson-2021 10	Case report	1	Tracer not stated	51	Dopamine transporter SPECT showed abnormal dopamine uptake bilaterally	This case highlights an atypical presentation of HD with parkinsonism and the need for specialized genetic testing for repeat expansion disorders
Mulroy-2020 11	Case report	1	Tracer not stated	52 *HDL-2	Bilateral asymmetric reduction in striatal uptake more in the right putamen	DAT-SPECT imaging can be abnormal in HDL-2
Chun-2022 12	Case report	1	[ ^18^ F]-FP-CIT PET/CT [ ^99^ ᵐTc]-ECD SPECT	18/43	** [ ^18^ F]-FP-CIT PET/CT ** showed reduced uptake in the basal ganglia, frontal, and parietotemporal lobes, with decreased DAT binding in the ventral and posterior putamen. [ ^99^ ᵐTc]-ECD SPECT revealed reduced perfusion in the basal ganglia, frontal, and temporal lobes	A multimodal imaging approach is recommended for tracking HD progression since no single technique is optimal. A single-tracer, dual-phase ^18^ F-FP-CIT PET test may help differentiate HD while reducing costs and radiation exposure

Abbreviations: HD, Huntington's disease; SPECT/CT, single-photon emission computed tomography–computed tomography.

DaTSCAN findings may be variable. Gamez et al. observed reduced [ ^123^ I]-FP-CIT (DaTSCAN) uptake in 8 out of 12 HD patients based on visual analysis, with 4 showing abnormal semiquantitative results, suggesting presynaptic dopaminergic involvement. They also found no linear correlation with disease severity, but they found that the patients with severe symptoms had more severe reductions in radioligand uptake. 7 In a 2-year follow-up study, Gamez et al. noted progressive uptake reductions of [ ^123^ I]-FP-CIT in the caudate and putamen nuclei, with average annual declines of 5.8 and 9.6%, respectively. Similarly, Larson described a HD patient with abnormal dopamine uptake bilaterally. 10

Kiferle et al. also reported significantly reduced caudate, putamen, and overall striatal uptake in HD patients compared with controls, supporting potential presynaptic dysfunction. 8 Mulroy et al. proposed that striatal atrophy could account for the reduced DaT uptake, aligning with the observations of Gamez et al. and Kiferle et al., suggesting that structural degeneration may contribute to the DaTSCAN findings. 7 8 9 11 However, MRI findings in our case indicated global cerebral atrophy with no prominent striatal atrophy identified.

In contrast, Hwang et al. using both [ ^99^ ᵐTc] TRODAT-1 and [ ^123^ I] IBZM, reported reduced DAT binding in only one out of four HD patients and more prominent reductions in D2 receptor binding, suggesting predominant postsynaptic involvement. 6 They concluded that presynaptic component is usually not affected but could happen in very advanced disease.

Several studies have also contributed to our understanding of DAT imaging in HD using other modalities. Chun demonstrated that early-phase [ ^18^ F]-FP-CIT PET/CT perfusion imaging showed patterns similar to [ ^99^ ᵐTc]-ECD SPECT, whereas late-phase imaging revealed decreased DAT binding in the ventral and posterior putamen. Based on these findings, the study proposed that a dual-phase [ ^18^ F]-FP-CIT PET protocol may serve as a single-tracer alternative for assessing both perfusion and dopaminergic function in HD. 12 Other PET radioligands have been developed to target key aspects of HD pathology, including microglial activation, C-11 β-CIT and phosphodiesterase 10A (PDE10A) expression. 5 13

The possibility of concomitant HD and Parkinson's disease (PD), as described in a case by Hadden et al., could also explain the imaging findings. 14 The lack of documented family history and possible antipsychotic-induced effects added to the diagnostic uncertainty. Early antipsychotic use led to parkinsonism-like symptoms, as managing HD chorea by reducing dopaminergic transmission can worsen involuntary movements and induce drug-related parkinsonism.

## Conclusion

Abnormal DaTSCAN does not necessarily indicate degenerative parkinsonism. Other neurodegenerative conditions, such as HD, should be considered, especially when clinical symptoms are unclear, and family history is undocumented. This case underscores the importance of increased awareness of HD presenting with abnormal DaTSCAN findings.

Further comparative studies are needed to correlate genetic findings, MRI, disease severity, and DaTSCAN results. While genetic testing remains the definitive diagnostic tool for HD, DaTSCAN may serve as a complementary modality to evaluate presynaptic dopaminergic involvement and potentially monitor disease progression. Future comparative studies are essential to correlate genetic findings, MRI, disease severity, and DaTSCAN results.
